# Temporal Modulation Enhances Medial Olivocochlear Reflex Efficacy: A TEOAE Suppression Study With Amplitude‐Modulated Broadband Noise

**DOI:** 10.1002/brb3.71665

**Published:** 2026-08-12

**Authors:** İrem Sendesen, İrem Karakuluk

**Affiliations:** ^1^ Audiology Department, Faculty of Health Science Gazi University Ankara Turkey; ^2^ Audiology Department, Faculty of Health Science Atılım University Ankara Turkey

**Keywords:** amplitude modulation, auditory efferent system, frequency specificity, medial olivocochlear reflex, TEOAE suppression

## Abstract

**Introduction:**

The medial olivocochlear reflex (MOCR), an efferent feedback pathway, modulates cochlear gain to enhance signal detection in noise. It is commonly assessed non‐invasively via contralateral suppression of transient‐evoked otoacoustic emissions (TEOAEs). While unmodulated noise is a standard elicitor, ecologically relevant amplitude‐modulated (AM) noises may be more effective activators, though literature findings are conflicting regarding the optimal modulation rate. This study aimed to compare the efficacy of unmodulated white noise (WN) and WN modulated at 10, 50, and 100 Hz (AM‐BBN) in eliciting the MOCR, as measured by TEOAE suppression. A secondary objective was to investigate the frequency specificity and laterality of the suppression effect.

**Methods:**

Forty‐six normal‐hearing adults participated. TEOAEs were recorded ipsilaterally while presenting contralateral noise stimuli (WN, M10, M50, and M100 Hz) at 60 dB SPL. Suppression was measured across frequency bands (1, 1.5, 2, 3, and 4 kHz) and ears. Non‐parametric statistical analyses were employed due to non‐normal data distribution.

**Results:**

A significant main effect was found for stimulus type (χ^2^(3) = 28.45, *p* < 0.001). Post‐hoc analyses revealed that M50 and M100 Hz elicited significantly greater suppression than WN (*p* = 0.003 and *p* = 0.007, respectively) and M10 Hz. Suppression was strongest at lower frequencies (1–2 kHz) and weakest at 4 kHz (*p* < 0.001). The right ear showed significantly greater suppression than the left (*p* = 0.003). No gender differences were observed.

**Conclusion:**

AM noises at moderate to high rates (50–100 Hz) are more effective MOCR activators than unmodulated noise. The efferent system demonstrates clear frequency‐specific and lateralized efficacy, with maximal suppression at lower frequencies and in the right ear. These findings underscore the importance of the temporal characteristics of contralateral stimuli and suggest optimized MOCR assessment using ecologically relevant, fluctuating sounds.

## Graphical abstract

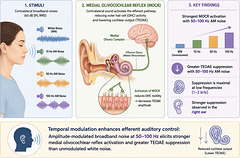

## 1 Introduction

The auditory efferent system, originating in the brainstem and projecting to the cochlea, provides a feedback mechanism for the central modulation of peripheral auditory processing. The most well‐known component of this system is the olivocochlear bundle (OCB), which consists of medial and lateral pathways (Warr 1980). The medial olivocochlear (MOC) system, in particular, projects predominantly to the outer hair cells (OHCs) and is implicated in several important functions, including sound localization, auditory attention, improved auditory sensitivity, signal detection in noise, protection against loud sounds, and reduction of masking effects (Bruel et al. 2001).

A key method for assessing MOC function in humans is the medial olivocochlear reflex (MOCR), which can be measured non‐invasively through the suppression of otoacoustic emissions (OAEs) (Guinan Jr 2018; Lopez‐Poveda 2018). Notably, transient‐evoked OAEs (TEOAEs)‐based contralateral suppression has been validated as one of the most reliable and sensitive methods for assessing MOC reflex function, demonstrating consistent results across diverse populations and test conditions (Mishra and Dinger 2016). TEOAEs, sounds generated by the cochlea in response to a brief acoustic stimulus, are reduced in amplitude when sound is presented to the contralateral ear. This contralateral suppression is believed to reflect MOC‐induced inhibition of OHC motility, which reduces cochlear gain (Collet et al. 1990; Veuillet et al. 1991).

Various contralateral suppressors have been used to elicit this reflex, including white noise (WN), narrowband noise, and speech noise. While unmodulated WN is a common elicitor, amplitude‐modulated (AM) noises are more frequently encountered in everyday acoustic environments. It has been hypothesized that AM noises might be more effective activators of the MOCR system due to this ecological relevance (Kalaiah et al. 2017). However, the literature on the effect of different AM rates on the MOCR is scarce and presents conflicting evidence. Some studies report similar suppression for WN and AM‐BBN (Boothalingam et al. 2014), while others found significant MOC suppression for 100 Hz AM‐BBN (Maison et al. 1999), and yet another reported maximal suppression for unmodulated noise with no special effect for 100 Hz modulation (Backus 2005).

Importantly, the source of these discrepancies remains unresolved and may stem from methodological differences in how cochlear activity is probed. For instance, Boothalingam et al. (2014) investigated the potential interaction between the 50 Hz stimulus rate and AM modulation but found no enhancement of suppression using stimulus‐frequency otoacoustic emissions (SFOAE) and tone‐burst OAEs (TBOAE). They suggested that the MOC system might simply integrate total energy rather than following rapid modulations. However, SFOAEs and TBOAEs probe restricted, frequency‐specific regions of the cochlea. In contrast, studies reporting an “AM advantage” often utilized TEOAEs, which are elicited by click stimuli that simultaneously activate a wide expanse of the basilar membrane (Maison et al. 1997). We hypothesize that the MOC system's enhanced response to AM cues may rely on pooling efferent inhibitory effects across multiple auditory filters (critical bands). Therefore, TEOAEs, which capture the aggregate response from these distributed cochlear generators, might be more sensitive to this widespread efferent activation than frequency‐specific assays.

Furthermore, regarding the frequency specificity of the reflex, early anatomical descriptions focused on bundle thickness; however, more recent immunohistochemical analyses in humans have provided valuable insights into the innervation density of the MOC system. Liberman and Liberman (2019) demonstrated that although MOC innervation is generally sparse in humans, the density of efferent terminals peaks in the mid‐cochlear regions. This anatomical distribution is of particular interest given that OAE suppression is typically observed to be greater at low‐to‐mid frequencies compared to high frequencies, suggesting a direct link between innervation density and reflex strength (Andrade et al. 2017; Maison et al. 1999).

Therefore, the present study was designed to go beyond a simple evaluation of elicitors by testing the hypothesis that the MOCR sensitivity to AM stimuli is better captured through broadband TEOAE monitoring. Specifically, we aimed to compare the efficacy of unmodulated WN and WN modulated at 10, 50, and 100 Hz (AM‐BBN)—rates selected to align with the putative peak sensitivity of MOC neurons (Gummer et al. 1988)—in eliciting the MOCR. By revisiting the interaction between modulation rates and the efferent system using a broadband TEOAE paradigm, this study aims to clarify whether the previously reported lack of AM enhancement extends to clinical TEOAE measures or if capturing the simultaneous efferent modulation across multiple cochlear frequency regions reveals a distinct sensitivity to ecologically relevant modulations (Boothalingam et al. 2014). A secondary objective was to investigate the frequency specificity and laterality of the suppression effect. Understanding these parameters is important for developing more ecologically valid and sensitive clinical assessments of efferent function.

## 2 Materials and Methods

### 2.1 Participants

A total of 46 young adults (22 males, 24 females; mean age = 24.3 ± 3.1 years) with normal hearing participated. Normal hearing was defined as pure‐tone air conduction thresholds of ≤ 20 dB HL at octave frequencies from 250 to 8000 Hz, consistent with criteria used in studies of efferent function (Gafoor and Uppunda 2024; Wojtczak et al. 2019). All participants had normal, type A tympanograms bilaterally, indicating normal middle ear function, and no history of auditory pathology, neurological disorders, or ototoxic medication use. Written informed consent was obtained from all participants prior to the study, which was approved by the institutional ethics committee.

### 2.2 Stimuli and Procedure

The experiment was conducted in a sound‐attenuated booth. TEOAEs were recorded from both ears using an OAE system (Otodynamics Ltd.), following established protocols for measuring efferent effects (Backus and Guinan 2006; Gafoor and Uppunda 2024). The ipsilateral stimulus consisted of a non‐linear click sequence presented at 60 dB peSPL. The click duration was 80 µs, and they were presented at a rate of 50 s^−1^. For each recording condition, a minimum of 260 sweeps were collected and averaged after online artifact rejection. A TEOAE response was considered present and valid for analysis if the overall response SNR exceeded 6 dB in the baseline (no suppressor) condition. While the literature emphasizes that higher SNR criteria, such as > 15 to 20 dB, are optimal for distinguishing small MOC effects from noise floor pollution (Goodman et al. 2013; Mishra and Lutman 2013), the > 6 dB threshold utilized in the present study reflects a standard clinical limit rather than a strict experimental inclusion criterion. This clinical threshold, combined with stringent artifact rejection and repeatability controls, was considered adequate to capture the relative MOC‐induced suppression effects in this cohort. The probe was sealed acoustically in the ear canal to ensure stability. Probe fit stability was monitored continuously throughout the recording session. The “Stimulus Stability” index provided by the ILO88 software was required to exceed 90%, and the stimulus waveform was visually inspected for any changes in amplitude or morphology that would indicate a probe drift or pressure leak. If a shift in stimulus level exceeding ±1 dB was observed, the recording was halted, and the probe was refitted. TEOAEs were analyzed in a time window excluding the initial stimulus artifact.

Four types of contralateral acoustic stimuli (CAS) were generated using Praat software and delivered to the contralateral ear via an insert phone (Etymotic Research ER‐3A) at 60 dB SPL (RMS), a level chosen to minimize the potential activation of the middle‐ear muscle reflex (MEMR) (Gafoor and Uppunda 2024; Jennings 2021): Unmodulated WN, AM WN at 10 Hz (M10 Hz; 100% modulation depth), AM WN at 50 Hz (M50 Hz; 100% modulation depth), and AM WN at 100 Hz (M100 Hz; 100% modulation depth). All stimuli were calibrated to have equal RMS levels to ensure consistent total energy delivery across conditions.

The order of presentation of the four stimulus conditions was randomized across participants to control for order effects.

### 2.3 Data Acquisition

For each participant and condition, TEOAEs were recorded both in silence (baseline) and in the presence of the contralateral suppressor. The suppression magnitude was calculated as the difference in TEOAE amplitude (in dB) between the baseline and suppressor conditions. This was done for the overall response and for five half‐octave frequency bands centered at 1, 1.5, 2, 3, and 4 kHz.

### 2.4 Data Analysis

Statistical analyses were performed using R software (version 4.3.1). The Shapiro‐Wilk test and Levene's test were used to assess the normality of distribution and homogeneity of variances, respectively. As the data violated parametric assumptions, non‐parametric tests were employed.

The Friedman test, a non‐parametric alternative to repeated‐measures ANOVA, was used to assess the within‐subjects effects of the main factors: Stimulus Type (4 levels: WN, M10, M50, and M100 Hz), Frequency (5 levels: 1, 1.5, 2, 3, and 4 kHz), and Ear (2 levels: left and right). For significant main effects, post‐hoc pairwise comparisons were conducted using the Dunn test with Bonferroni correction to control for multiple comparisons. The Mann–Whitney *U* test was used to investigate potential differences in MOCR strength between genders. Effect sizes for Friedman tests were reported using Kendall's W. The alpha level for statistical significance was set at *p* < 0.05.

## 3 Results

The present study investigated the differential effects of unmodulated and AM broadband noises on the activation of the MOCR, as quantified by contralateral suppression of TEOAEs. The analysis employed non‐parametric statistical tests due to the non‐normal distribution of the data.

### 3.1 Descriptive Statistics

The descriptive statistics for the mean TEOAE suppression values, stratified by stimulus type, frequency, and ear, are comprehensively presented in Table 1, providing a detailed overview of the data underlying the statistical conclusions.

**TABLE 1:** Summary of mean response values and standard deviations by frequency, stimulus type, and ear.

Frequency (kHz)	Stimulus type	Right ear (dB) (mean ± SD)	Left ear (dB) (mean ± SD)	Bilateral average (dB) (mean ± SD)
**1**	WN	2.34 ± 2.42	1.08 ± 2.69	1.71 ± 2.58
**1**	M10 Hz	1.91 ± 2.69	1.26 ± 2.50	1.59 ± 2.60
**1**	M50 Hz	2.42 ± 2.71	1.31 ± 2.47	1.87 ± 2.62
**1**	M100 Hz	2.39 ± 2.67	1.30 ± 2.51	1.85 ± 2.61
**1.5**	WN	2.14 ± 2.15	1.63 ± 2.61	1.89 ± 2.41
**1.5**	M10 Hz	2.22 ± 2.49	1.75 ± 2.52	1.99 ± 2.51
**1.5**	M50 Hz	2.44 ± 2.55	1.86 ± 2.60	2.15 ± 2.58
**1.5**	M100 Hz	2.34 ± 2.56	1.79 ± 2.59	2.07 ± 2.58
**2**	WN	1.58 ± 2.39	1.36 ± 2.37	1.47 ± 2.38
**2**	M10 Hz	1.61 ± 2.56	1.39 ± 2.48	1.50 ± 2.52
**2**	M50 Hz	1.71 ± 2.60	1.45 ± 2.53	1.58 ± 2.57
**2**	M100 Hz	1.69 ± 2.57	1.41 ± 2.49	1.55 ± 2.53
**3**	WN	1.08 ± 2.41	0.63 ± 2.39	0.86 ± 2.40
**3**	M10 Hz	1.14 ± 2.43	0.80 ± 2.47	0.97 ± 2.45
**3**	M50 Hz	1.21 ± 2.51	0.88 ± 2.48	1.05 ± 2.50
**3**	M100 Hz	1.19 ± 2.50	0.84 ± 2.45	1.02 ± 2.48
**4**	WN	0.95 ± 2.45	0.44 ± 2.28	0.70 ± 2.38
**4**	M10 Hz	1.02 ± 2.41	0.59 ± 2.46	0.81 ± 2.44
**4**	M50 Hz	1.11 ± 2.50	0.64 ± 2.43	0.88 ± 2.47
**4**	M100 Hz	1.07 ± 2.46	0.61 ± 2.41	0.84 ± 2.44

*Note*: The combined standard deviation for both ears was calculated using the formula for pooling standard deviations of two independent samples.

Abbreviations: M10 Hz, 10 Hz modulation; M100 Hz, 100 Hz modulation; M50 Hz, 50 Hz modulation; WN, white noise.

### 3.2 Effect of Contralateral Stimulus Type

A highly significant main effect was observed for the stimulus type (*χ* ^2^(3) = 28.45, *p* < 0.001, Kendall's *W* = 0.21), indicating that the efficacy of the MOCR was not uniform across the different noise conditions. Post‐hoc pairwise comparisons, detailed in Table 2, elucidated the nature of these differences.

**TABLE 2:** Post hoc comparisons for stimulus type (Dunn Test) according to average of both ears.

Comparison	*Z*	*p*	*p*_adjusted_	Effect size (*r*)
**M50 Hz vs. WN**	3.42	0.003	0.018	0.50
**M100 Hz vs. WN**	3.18	0.007	0.028	0.47
**M50 Hz vs. M10 Hz**	2.78	0.021	0.042	0.41
**M100 Hz vs. M10 Hz**	2.56	0.038	0.076	0.38

Abbreviations: M10 Hz, 10 Hz modulation; M100 Hz, 100 Hz modulation; M50 Hz, 50 Hz modulation; WN, white noise.

**FIGURE 1:**
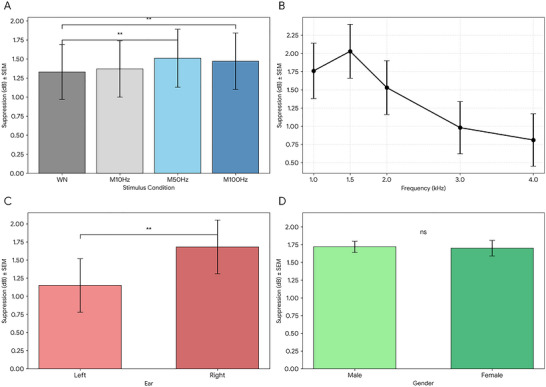
Characteristics of medial olivocochlear reflex (MOCR) suppression across different experimental conditions. To illustrate the main statistical effects, data in each panel represent values averaged across the non‐target variables. (A) Effect of Stimulus Type: Mean overall suppression levels for unmodulated (WN) and AM (M10, M50, and M100 Hz) WN, calculated by averaging the responses across all frequency bands and both ears Asterisks indicate statistically significant differences compared to WN after Bonferroni correction (**p < 0.01). (B) Frequency Specificity: The magnitude of suppression across the frequency range (1–4 kHz), averaged across all four contralateral stimulus conditions and both ears. (C) Laterality: Comparison of suppression between the left and right ears, averaged across all stimulus types and frequency bands, showing a significant right‐ear advantage (**p < 0.01). (D) Gender: Comparison of MOCR strength between male and female participants, averaged across all stimulus types, frequencies, and ears (ns: non‐significant). Error bars represent the standard error of the mean (SEM).

Specifically, WN AM at 50 Hz (M50 Hz) and 100 Hz (M100 Hz) elicited significantly greater TEOAE suppression (Figure 1A) compared to unmodulated WN (*p* = 0.003 and *p* = 0.007, respectively). Furthermore, the suppression elicited by M50 Hz was statistically greater than that produced by the 10 Hz modulated noise (M10 Hz) (*p* = 0.042). The comparison between M100 Hz and M10 Hz did not survive correction and was considered a non‐significant trend (*p* = 0.076). Importantly, the direct comparison between the two higher modulation rates, M50 Hz and M100 Hz, was also conducted and revealed no statistically significant difference in suppression magnitude (*p* = 0.688). Finally, the suppression produced by unmodulated WN and M10 Hz did not differ significantly from each other. Effect sizes (*r* = *Z*/√*N*) for significant comparisons were as follows: M50 Hz versus WN (*r* = 0.50), M100 Hz versus WN (*r* = 0.47), and M50 Hz versus M10 Hz (*r* = 0.41).

### 3.3 Effect of Frequency

The analysis uncovered a robust main effect for frequency (*χ* ^2^(4) = 35.12, *p* < 0.001, Kendall's *W* = 0.19), demonstrating that the magnitude of contralateral suppression was frequency‐dependent. Post‐hoc tests confirmed that suppression was most effective in the lower frequency regions (Figure 1B), with the greatest effect observed at 1 kHz. This suppression effect exhibited a progressive and significant decline towards the higher frequencies, with the weakest suppression measured at 4 kHz (*p* < 0.001 for the comparison between 1 and 4 kHz).

### 3.4 Effect of Ear (Laterality)

A significant main effect for the ear was identified (*χ* ^2^(1) = 8.76, *p* = 0.003, Kendall's *W* = 0.19). The analysis demonstrated that the right ear consistently exhibited a greater magnitude of contralateral suppression across all stimulus conditions and frequencies compared to the left ear (Figure 1C) (*p* = 0.003).

### 3.5 Analysis of Gender Differences

An analysis of potential gender differences in MOCR strength was conducted. A Mann–Whitney *U* test indicated no statistically significant difference in suppression measures between male and female participants (Figure 1D) (*U* = 210.5, *p* = 0.187), suggesting that the function of the MOC efferent system, as measured by this paradigm, is not sexually dimorphic in this cohort of normal‐hearing adults.

## 4 Discussion

The present study demonstrated that the temporal structure of a contralateral broadband noise significantly influences its efficacy in activating the MOCR, as quantified by the suppression of TEOAEs. Furthermore, the results revealed a pronounced frequency‐specific and lateralized pattern of suppression. The following discussion integrates these findings with the existing literature to elucidate the mechanisms and functional implications of the MOCR.

### 4.1 Efficacy of AM Noises in MOCR Activation

The key finding of this study is that amplitude‐modulated broadband noises (AM‐BBN), particularly at 50 and 100 Hz, elicited significantly greater TEOAE suppression than unmodulated WN. This result aligns with a body of evidence suggesting that the MOCR is sensitive to temporally fluctuating stimuli, which are characteristic of most natural sounds, including speech and environmental noises (Lopez‐Poveda 2018; Micheyl and Collet 1996). The enhanced efficacy of modulated stimuli can be explained by the neural encoding properties of the auditory efferent pathway. Gummer et al. (1988) recorded from MOC neurons in guinea pigs and found that their modulation transfer functions (MTFs) showed a peak sensitivity around 100 Hz. This suggests that the MOC system is physiologically tuned to respond most strongly to amplitude fluctuations in this frequency range. To reconcile the seemingly contradictory findings in the literature—specifically why the present study observed an “AM advantage” while Boothalingam et al. (2014) and Mertes (2018) did not—we propose a hypothesis based on the synergistic interplay between broadband spatial integration and temporal synchrony.

First, regarding the OAE type, while we acknowledge that both TEOAEs and SFOAEs primarily arise from linear coherent reflection mechanisms, they differ fundamentally in their monitoring scope. We hypothesize that the broadband nature of the ipsilateral click stimulus itself is integrated by the MOCR circuit. Unlike SFOAEs, which utilize a narrowband probe that provides only localized ipsilateral excitation, the click simultaneously activates a wide expanse of the basilar membrane. We postulate that this broad, synchronous ipsilateral input combines with the contralateral AM noise to provide a stronger drive to the MOC system, thereby facilitating the efferent network's ability to track the temporal modulation envelope. The lack of AM enhancement in the study by Boothalingam et al. (2014) may therefore be attributed to the narrowband nature of their SFOAE/TBOAE probes, which might have missed the cumulative effect of modulation‐specific suppression.

Second, regarding the probe repetition rate, we acknowledge that the 50 clicks/s rate used in our study is high enough to elicit the ipsilateral MOCR. Physiologically, ipsilateral activation reduces the baseline cochlear gain, potentially leading to a saturation effect that would theoretically reduce the dynamic range available for observing additional contralateral suppression. However, the fact that we observed enhanced suppression despite this potential saturation points to a distinct facilitatory mechanism unique to the 50 Hz rate. We propose that the 50 Hz click train (20 ms inter‐click interval) creates a temporal template that synchronizes with the periodicity of the 50 Hz (20 ms) and 100 Hz (10 ms) AM envelopes. Supported by evidence that cochlear nucleus neurons phase‐lock robustly to AM envelopes, we postulate that this rhythmic alignment creates a “temporal entrainment” within the efferent feedback loop (Frisina et al. 1990). This synchrony likely maximizes the drive to MOC neurons, overriding the depressive effects of ipsilateral saturation.

Crucially, this hypothesis explains the divergence from Mertes (2018), who utilized broadband TEOAEs but employed a stimulus rate of ∼20/s. While the slower rate minimized ipsilateral saturation, it also eliminated the temporal synchrony with the 100 Hz modulation. Without the facilitatory effect of rate entrainment, the broadband integration alone was likely insufficient to drive the AM advantage. Therefore, our findings suggest that the AM enhancement is contingent upon a specific combination: broadband spatial integration and temporal rate synchrony.

It is important to address the potential influence of stimulus peak levels. Since all stimuli were equated for RMS intensity (60 dB SPL), the AM conditions inherently contained higher instantaneous peaks than the unmodulated WN. One might argue that the increased suppression was driven by these higher peaks rather than the modulation itself. However, our results argue against this “peak‐level” hypothesis. The M10 Hz stimulus, which shared the same high‐peak characteristics as the M50 and M100 Hz stimuli, failed to produce significantly greater suppression than unmodulated noise. This dissociation indicates that the enhanced MOC activity observed at 50 and 100 Hz is driven by the specific temporal rates that align with the system's tuning, rather than by intensity peaks.

While the absolute increase in suppression for AM stimuli was modest (∼0.2 dB), the highly structured nature of the data—specifically the consistent frequency tuning (peaking at 1–2 kHz) and the robust right‐ear advantage across the cohort—strongly indicates that this enhancement reflects a genuine physiological mechanism rather than stochastic measurement instability. Random noise floor fluctuations would not produce such systematic, anatomically consistent patterns.

### 4.2 Frequency Specificity of the Suppression Effect

A consistent finding in our study was the strong dependence of suppression on frequency, with the greatest effect observed at 1 kHz and a progressive decline towards higher frequencies (4 kHz). This low‐frequency bias in MOCR efficacy is a well‐replicated phenomenon (Lilaonitkul and Guinan Jr 2009; Zhao and Dhar 2012). Historically, this distribution was considered paradoxical given early reports suggesting that the OCB is thicker in the basal (high‐frequency) region of the cochlea. However, recent immunohistochemical findings by Liberman and Liberman (2019) have resolved this discrepancy by characterizing the MOC innervation density in humans. They demonstrated that the density of MOC terminals is actually highest in the mid‐cochlear regions (corresponding to the 1–3 kHz range) rather than the base. This anatomical evidence directly aligns with our functional findings, where maximal suppression was observed at 1–2 kHz, suggesting that the strength of the MOC reflex is constrained by the density of effector synapses on the OHCs.

This frequency specificity is best explained by the tonotopic organization of the efferent pathways and their connectivity with the cochlea. The MOC neurons that project to a specific cochlear region are most effectively activated by sound frequencies close to the characteristic frequency of that region (Lilaonitkul and Guinan Jr 2009; Zhao and Dhar 2012). The TEOAE response, particularly in the 1–2 kHz range, is often the most robust and stable in normal‐hearing ears. Therefore, the suppression effect is most easily measured and is largest in this frequency region. Furthermore, physiological studies suggest that the antimasking benefits of the MOCR may be most advantageous in the low‐frequency regions where phase‐locking occurs, aiding in the detection of signals in noise (Lopez‐Poveda 2018; Micheyl and Collet 1996). This finding is consistent with reviews highlighting that the efferent system's modulation of cochlear response is frequency‐dependent (Guinan Jr 2018; Lopez‐Poveda 2018).

Additionally, methodological factors related to the recording parameters may have contributed to the reduced suppression observed at 4 kHz. The present study employed a non‐linear click paradigm, which necessitates the windowing of the initial response to eliminate stimulus artifact. As highlighted by Goodman et al. (2021), high‐frequency TEOAE components typically exhibit shorter latencies. Consequently, the exclusion of the initial time window to avoid artifacts likely resulted in the loss of short‐latency components, particularly at 4 kHz. Although the MOC effect is reportedly smaller for short‐latency versus long‐latency components, its exclusion may have led to an underestimation of the suppression effect in the high‐frequency range in this study.

### 4.3 Laterality: Asymmetry in MOCR Function

We observed a significant lateralization effect, with the right ear demonstrating consistently greater contralateral suppression across all conditions than the left ear. This finding supports the growing evidence for functional asymmetry in the human auditory system, particularly in efferent processing (Bianchi et al. 2013). However, this asymmetry cannot be attributed to differences between crossed and uncrossed pathway strengths, as contralateral suppression is mediated primarily by the crossed medial OCB in both ears. Instead, the observed right‐ear advantage likely reflects the asymmetry in top‐down corticofugal modulation. Studies in animals and humans have suggested that descending inputs from the auditory cortex play an important role in modulating olivocochlear activity (Acuña et al. 2023; Bianchi et al. 2013).

This functional lateralization is frequently linked to the hemispheric specialization for processing different acoustic features, with the right ear (left hemisphere) often dominant for processing rapid temporal transitions found in speech. The stronger MOCR in the right ear could be part of a neural network optimized for extracting signals from noisy environments, a core function of the efferent system (Lopez‐Poveda 2018; Micheyl and Collet 1996). Interestingly, this finding aligns with observations in specialized populations like musicians, who often show enhanced efferent function, though the relationship between laterality and musical training remains an area for further exploration (Acuña et al. 2023; Morlet et al. 2003).

### 4.4 Lack of Gender Differences

The analysis revealed no statistically significant difference in MOCR strength between male and female participants. This suggests that the basic functional properties of the MOC efferent system, as measured by TEOAE contralateral suppression, are not sexually dimorphic in young, normal‐hearing adults. This finding allows for the pooling of data across genders in studies investigating MOCR function in similar populations, simplifying recruitment and analysis.

### 4.5 Functional and Clinical Implications

The finding that AM noises are sensitive MOCR activators has significant functional implications. The acoustic environment is replete with temporally fluctuating sounds. Our findings more precisely highlight the underlying physiological capacity of the MOCR to track and integrate temporal envelopes when optimally driven. This suggests the efferent system possesses the necessary neural tuning to process fluctuating maskers, even if its activation in unconstrained, real‐world situations relies on more complex and less perfectly synchronized acoustic inputs. By reducing cochlear gain in a dynamic manner, synchronized to the envelope of background noise, the MOCR could enhance the neural representation of target signals (e.g., a speaker's voice) against a fluctuating masker, thereby improving speech intelligibility in noise (Lopez‐Poveda 2018; Micheyl and Collet 1996).

However, a recent systematic review and meta‐analysis by Gafoor and Uppunda (2024) suggests caution in overinterpreting the perceptual benefits of MOCR. Their analysis of 21 studies found that the MOCR strength, as measured by contralateral suppression of OAEs, accounted for less than 1% of the variance in speech perception in noise among neurotypical individuals. This indicates that while the MOCR may be robustly activated by certain stimuli, its direct contribution to complex listening tasks like speech perception in noise may be limited or obscured by other factors. This discrepancy highlights the possibility that OAE‐based measures may not fully capture the functional role of the MOCR or that its benefits are highly task‐ and context‐dependent. This conclusion is further supported by a subsequent linear mixed‐effects modeling study, which found no significant contribution of MOCR strength (via TEOAE suppression) to speech perception in noise across various stimuli, SNRs, metrics, and age groups (Gafoor and Uppunda 2024).

From a clinical perspective, while our findings suggest that AM noises can be effective MOCR elicitors under specific test conditions, the mixed results in the literature advise against definitive clinical recommendations at this stage. These findings underscore the importance of carefully selecting the contralateral stimulus when assessing MOCR function. Using an unmodulated noise may underestimate the full efferent potential of an individual. Further research is needed to standardize protocols and establish normative data before a standardized stimulus with proven ecological validity, such as a 50 or 100 Hz AM noise, could be recommended as a more sensitive and functionally relevant measure of efferent integrity. This could be particularly useful in assessing populations suspected of having efferent dysfunction, such as individuals with auditory processing disorders, tinnitus, or difficulty hearing in noise despite normal pure‐tone thresholds (Guinan Jr 2018; Lopez‐Poveda 2018). The observed enhanced efferent function in musicians Acuña et al. (2023) also suggests that MOCR assessment with optimized stimuli could be a valuable tool for investigating experience‐dependent plasticity in the auditory system.

### 4.6 Limitations and Future Directions

This study was limited to young adults with normal hearing. Future research should investigate whether these findings generalize to older populations and individuals with hearing impairment, where efferent function may be altered. Furthermore, while AM noise was used, other ecologically valid stimuli like speech babble or real‐world noises (e.g., cafeteria noise) could be explored to better understand MOCR function in everyday life (Lopez‐Poveda 2018). Finally, employing other OAE types (e.g., DPOAEs) and physiological measures could help reconcile the discrepancies in the literature regarding the MOCR's sensitivity to amplitude modulation.

A significant limitation of this study is the absence of individual MEMR threshold measurements for each modulated stimulus condition. Although the 60 dB SPL RMS level was chosen to remain below typical clinical MEMR thresholds, AM noises inherently contain higher instantaneous peaks than unmodulated noise at the same RMS level. MEMR thresholds can be lower for modulated stimuli, raising the possibility that the higher peaks in our M50 and M100 Hz conditions may have triggered subtle, frequency‐dependent middle‐ear mechanical changes (Feeney and Keefe 2001; Mertes 2018). While the frequency‐specific suppression pattern we observed (strongest at 1–2 kHz, weakest at 4 kHz) is more consistent with MOC‐mediated efferent inhibition than with the broad low‐frequency attenuation typically associated with MEMR activation (Maison et al. 1997), we cannot definitively rule out a partial contribution from the middle‐ear muscle reflex. Future studies should incorporate wideband acoustic immittance or individual MEMR threshold measurements for each stimulus to disentangle MOC and MEM contributions.

It is also important to note that the perceptual benefits of MOCR activation may not be directly observable in all behavioral tasks. For instance, Wojtczak et al. (2019) found that while noise precursors improved the detection of amplitude modulation (AM) in noise, simultaneous measurements of stimulus‐frequency otoacoustic emissions (SFOAEs) revealed that this improvement was not accompanied by significant MOCR effects on cochlear responses for elicitor levels that produced behavioral unmasking. This suggests that the large AM unmasking effects may arise primarily from post‐cochlear auditory processing rather than from MOCR‐induced changes in cochlear gain. This finding aligns with the meta‐analytic results and the experimental findings of Gafoor and Uppunda (2024), indicating that the functional role of the MOCR might be more nuanced than a simple gain reduction mechanism. Therefore, future studies should aim to integrate physiological MOCR measures with behavioral tasks to better understand the conditions under which efferent activation translates to perceptual benefits and to explore potential dissociations between robust physiological measures of MOCR strength and behavioral outcomes like speech perception in noise.

Finally, a significant methodological limitation concerns the reporting of individual signal‐to‐noise ratios (SNRs). We agree with recent literature advocating that high SNRs, ideally approaching 20 to 25 dB, are crucial for reliably distinguishing small MOC effects from the noise floor (Goodman et al. 2013; Mishra and Lutman 2013). Unfortunately, due to an unexpected hardware failure of the clinical ILO system, the raw data logs containing the precise SNR values for each recording became permanently irretrievable. The exported dataset only retained the final calculated suppression magnitudes. However, to ensure signal fidelity in the absence of explicit SNR data, we strictly enforced a waveform reproducibility criterion of > 70%, with the vast majority of our recordings exceeding 80%. This high level of reproducibility, combined with tight artifact rejection and high stimulus stability (> 90%), serves as a robust surrogate metric for signal quality, ensuring that the background noise floor is sufficiently low. While we acknowledge the absence of detailed SNR reporting as a critical oversight, the stringent reproducibility criteria support the validity of the observed suppression patterns.

## 5 Conclusion

In conclusion, the present study demonstrates that AM‐BBN, particularly at modulation rates of 50 and 100 Hz, elicits statistically greater TEOAE suppression than unmodulated WN. However, it is important to contextualize this finding: the absolute increase in suppression was modest (∼0.2 dB). Given this small effect size and the intersubject variability, AM‐BBN may offer limited immediate practical advantage over standard WN for routine clinical applications.

The primary significance of these findings lies in their physiological implications rather than clinical superiority. The observation that suppression is maximized at specific modulation rates (50–100 Hz) provides functional evidence in humans that mirrors the MTFs described in animal models. This suggests that the human MOC system is not merely a gain control system driven by overall energy but is temporally tuned to envelope fluctuations characteristic of biologically relevant sounds. Thus, while AM‐BBN may not yet replace WN in standard test batteries, it represents a valuable experimental probe for investigating the temporal processing capabilities of the auditory efferent system.

## Author Contributions

**İrem Sendesen**: conceptualization, methodology, writing – original draft, writing – review and editing, formal analysis, supervision. **İrem Karakülük**: Methodology, investigation, data curation, writing – review and editing.

## Ethics Statement

All procedures performed in studies involving human participants were in accordance with the ethical standards of the Health Science Research Ethics Committee (protocol number/approval code: İ05‐312‐97) and with the 1964 Helsinki Declaration and its later amendments or comparable ethical standards.

## Consent

Informed consent was obtained from all individual participants included in the study.

## Funding

The authors have nothing to report.

## Conflicts of Interest

The authors declare no conflicts of interest.

## Data Availability

The data that support the findings of this study are available from the corresponding author upon reasonable request.
